# 3D bioprinting patient-specific grafts for tendon/ligament repair in motion: emerging trends and challenges

**DOI:** 10.3389/fbioe.2025.1643430

**Published:** 2025-08-22

**Authors:** Xuejian Bai, Yihan Yang, Jinwei Chu, Yang Deng, Mingwei Li, Huaiyu Yang

**Affiliations:** ^1^ The Third Department of Orthopedic Surgery, Fuxin Mining General Hospital of Liaoning Health Industry Group, Liaoning, China; ^2^ Qingdao Film Academy, Qingdao, China

**Keywords:** biomechanics, personalized treatment, artificial intelligence, medical-engineering integration, sports injury

## Abstract

Tendon/ligament (T/L) injuries sustained during motion are highly prevalent and severely impact athletes’ careers and quality of life. Current treatments, including autografts, allografts, and synthetic ligaments, have limitations such as donor site morbidity, immune rejection, and biomechanical mismatch, especially under dynamic loading conditions encountered in motion. 3D bioprinting offers a revolutionary approach for constructing patient-specific T/L grafts. This Mini Review summarizes recent advancements in utilizing 3D bioprinting to fabricate patient-specific grafts for T/L repair, with a particular focus on strategies catering to the functional demands of “in motion” recovery. Key emerging trends in bioink development (balancing mechanical properties with bioactivity), cell selection and optimization, printing strategies (e.g., multi-material hierarchical printing, biomimetic design for complex mechanical loading), and post-printing maturation culture (e.g., multi-modal mechanical stimulation via bioreactors) are discussed. Furthermore, this review highlights critical challenges in the field, including precise matching and long-term maintenance of graft mechanical properties, effective vascularization and innervation, scalable manufacturing and quality control, and hurdles in clinical translation. Finally, this review underscores the immense potential of 3D bioprinting in personalized, functional T/L repair and envisions future research directions, such as the application of smart biomaterials and 4D bioprinting, refined *in vitro* maturation strategies, and *in vivo* bioprinting technologies, ultimately aiming to achieve robust tissue functional restoration “in motion.”

## 1 Introduction

Tendons and ligaments (T/Ls) are crucial connective tissues linking muscles to bones and bones to bones, respectively, essential for joint stability and locomotion (Tachibana et al., 2022). In competitive sports and high-intensity physical activities, T/L injuries such as anterior cruciate ligament (ACL) rupture (Gans et al., 2018), Achilles tendon rupture (Li et al., 2018), and rotator cuff tears are exceedingly common (Doi et al., 2019). These injuries not only cause significant pain and functional impairment but can also terminate athletic careers and substantially diminish quality of life. A specific challenge in repairing these injuries is that the healed tissue must withstand complex and variable dynamic mechanical loads encountered “in motion,” including high stress, high strain rates (Freedman and Mooney, 2019), and sustained fatigue loading, thereby placing stringent demands on the graft’s mechanical properties and biological integration (No et al., 2020).

Current clinical treatments for T/L ruptures primarily involve autografts (Valianatos et al., 2020), allografts (Renshaw et al., 2024), and synthetic grafts (Freedman and Mooney, 2019). Autografts (e.g., hamstring tendon or bone-patellar tendon-bone) eliminate immune rejection risks and possess good biological integration potential (Chen et al., 2025). However, their availability is limited, and harvesting can lead to donor site morbidity (e.g., pain, functional deficit), with graft size and shape often difficult to perfectly match the defect site. Allografts circumvent donor site issues but carry risks of immune rejection, disease transmission (Fishman et al., 2012; Fishman and Grossi, 2014), and potential degradation of biomechanical properties during sterilization and storage, with inflammatory responses potentially affecting long-term stability (Zhang et al., 2022). Synthetic grafts (e.g., polyester materials) can provide initial mechanical strength but are prone to long-term wear and tear, mechanical failure due to mismatch, and chronic inflammation due to suboptimal biocompatibility, lacking biological activity and integration capacity (Rohringer et al., 2023). The inherent limitations of these existing therapeutic modalities underscore the urgent need for novel and effective T/L repair strategies.

In recent years, 3D bioprinting has emerged as a revolutionary technology in tissue engineering and regenerative medicine (Chansoria and Shirwaiker, 2019). By precisely controlling the spatial arrangement of cells, biomaterials, and bioactive factors in three dimensions (Matai et al., 2020), this technology enables the fabrication of tissue/organ substitutes that possess complex anatomical structures and physiological functions (Murphy and Atala, 2014). While other advanced tissue engineering techniques, such as electrospinning, cell sheet engineering, and acellular matrix scaffolds, offer valuable approaches by mimicking specific aspects of native tissues (Xing et al., 2020), 3D bioprinting provides unparalleled advantages. For instance, while electrospinning excels at creating fibrous structures that effectively mimic the extracellular matrix, 3D bioprinting uniquely allows for the achievement of patient-specific macroscopic anatomical structures and precise three-dimensional spatial control over the placement and organization of cells, which is crucial for recreating complex tissue architectures and functions (Yi et al., 2021). Its immense potential in personalized medicine, particularly in manufacturing implants with patient-specific geometries, structures, and biological characteristics, offers new hope for T/L repair. By integrating patient-specific medical imaging data (e.g., MRI, CT), 3D bioprinting can design and manufacture T/L grafts that perfectly match the defect site, thereby promising better restoration of complex functions “in motion” (Wang et al., 2022). This mini review will focus on the latest advancements in 3D bioprinting for constructing patient-specific T/L grafts tailored for “in motion” demands. It will discuss emerging trends in key areas such as biomaterial and bioink innovation, cell source selection and optimization, structural biomimetic design strategies, and post-printing maturation techniques, while also deeply analyzing the major challenges and future prospects in this burgeoning field.

## 2 Key elements and emerging trends in 3D bioprinting of tendon/ligament grafts

**FIGURE 1:**
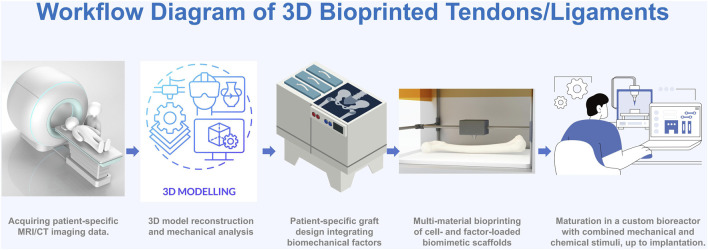
Workflow Diagram of 3D Bioprinted Tendons/Ligaments A schematic illustrating the process from patient imaging (MRI/CT), to 3D modeling and biomechanical analysis, patient-specific graft design, multi-material bioprinting, and maturation in a bioreactor, up to implantation.

Successful fabrication of 3D bioprinted tendon/ligament grafts hinges on the synergistic optimization of several key factors. Figure 1 illustrates the complete workflow, from patient-specific data acquisition to the culture of a mature construct ready for implantation. Key elements within this process, which have seen considerable progress and debate, include the choice of bioink, determination of the cell source, biomimetic graft design, and post-printing maturation strategies.

### 2.1 Innovation in bioinks and selection of cell sources

Bioinks play a vital role in 3D bioprinting, as their properties directly influence the feasibility of the printing process and the performance of the final biological structures. In recent years, researchers have made significant progress in developing and optimizing bioinks. For example, studies have shown that the rheological (referring to their flow and deformation properties), physical-mechanical, and biological functionalities of bioinks are key factors affecting their printability and cell viability (Derakhshanfar et al., 2018). Furthermore, the development of multi-component hydrogel bioink systems has opened up new possibilities for enhancing printing accuracy, shape fidelity, and biological functionality (Cui et al., 2020).

In the composition of bioinks, the combination of natural and synthetic materials has been shown to enhance the stability and biocompatibility of bioinks. For example, a composite bioink made from methyl methacrylate-modified xanthan gum and gelatin exhibits excellent shear thinning properties and biocompatibility, which makes it highly suitable for 3D bioprinting with superior printing performance and cell viability (Garcia-Cruz et al., 2021). Moreover, the introduction of nanocomposites offers a new approach to enhancing bioinks by improving their viscosity, printability, and biocompatibility, thus showing significant potential in bone and cartilage tissue engineering (Cai et al., 2022).

In practical applications, the optimization of bioinks involves not only material selection but also adjustments to printing parameters. For instance, studies have shown that by modifying the composition of bioinks and adjusting printing parameters, the shape fidelity and cell viability of printed embryonic stem cells can be significantly enhanced (Ouyang et al., 2016). Furthermore, the microscopic heterogeneity of bioinks has been shown to improve the quality of printed complex structures and cell viability, offering new strategies for high-resolution 3D printing (Maciel et al., 2023).

#### 2.1.1 Natural polymer-based bioinks

Natural polymers such as collagen, gelatin, alginate, silk fibroin, and dECM exhibit excellent biocompatibility, inherent cell recognition, and tissue-specific cues advantageous for T/L repair (Abou Neel et al., 2013; Raftery et al., 2016; Kannan et al., 2025). Research indicates that using natural materials like decellularized extracellular matrix (dECM) and collagen can significantly enhance the biocompatibility of bioinks (Abaci and Guvendiren, 2020), as these materials not only provide the biochemical signals necessary for cell attachment and growth but also mimic the microenvironment of natural tissues. Studies have shown that the rheological, physical-mechanical, and biological functionalities of bioinks are key factors affecting their printability and cell viability. To enhance the printability of bioinks, researchers have developed strategies, including using thermally reversible gelatin networks to temporarily stabilize the bioink (Ouyang et al., 2020). Furthermore, using decellularized matrix-free bioinks can provide a biochemical environment similar to that of natural tissues (Abaci and Guvendiren, 2020). However, significant batch-to-batch variation, difficulty in standardization, immunogenic potential, and relatively weak mechanical properties remain challenges. Despite superior biocompatibility, natural materials (especially dECM) still face challenges such as significant batch-to-batch variation (Hussein et al., 2016), difficulty in standardization, and potential immunogenicity (even after decellularization). Their relatively weak mechanical properties are also a concern (Nakamura et al., 2017). The choice of dECM source (allogeneic, xenogeneic) and optimization of decellularization methods significantly impact the final bioink’s performance and biological effects (Rieder et al., 2016; Kim et al., 2020; de Wit et al., 2023; El-Husseiny et al., 2023), representing a current research focus and point of contention. Some scholars believe that finely tuning dECM composition and degradation products could guide more specific cellular responses and tissue regeneration (Kim and Kim, 2020; Kim et al., 2020). Recent approaches to overcome these include compositing with other biomaterials (e.g., hyaluronic acid, chondroitin sulfate) and refining crosslinking methods (e.g., enzymatic, photo-crosslinking—a method using light to induce material solidification—) to improve mechanical strength and stability (Teixeira et al., 2012).

#### 2.1.2 Synthetic polymer-based bioinks

Synthetic polymers like polycaprolactone (PCL), polylactic acid (PLA), polyglycolic acid (PGA), and their copolymer polylactic-co-glycolic acid (PLGA) see widespread application in tissue engineering contexts that require structural support, owing to their advantageous mechanical properties, tunable degradation rates, and excellent processability. The appropriate mechanical strength and degradation rate are crucial for the application of bioinks; the mechanical strength must be sufficient to support the stability of printed structures, while the degradation rate should match the speed of tissue regeneration. By incorporating components such as nanocellulose and nano-hydroxyapatite, the mechanical properties of bioinks can be significantly enhanced, while maintaining high cell survival rates (Korkeamäki et al., 2025). Improving their inherent bio-inertness and imparting cell adhesiveness often involves combining them with natural hydrogels (such as gelatin or alginate) to create composite bioinks, or modifying their surfaces with cell adhesion peptides like the RGD sequence (Tavafoghi et al., 2020). Emerging synthetic polymers, including certain elastic polyurethanes or poly (glycerol sebacate) (PGS) (Jeffries et al., 2015), are also garnering increased attention due to mechanical properties that more closely mimic natural T/Ls.

Significant challenges and ongoing academic discussions pertain to synthetic polymers. A primary concern is their bio-inertness, which may limit effective cell adhesion and functional expression. Their degradation products, such as acidic byproducts, also pose a risk of triggering local inflammatory responses (da Silva et al., 2018). Academic discussions therefore significantly focus on how to precisely control their degradation rates to align with new tissue formation and how to enhance their biological performance through structural designs like porous architectures and material composites (Zhang et al., 2014). Some researchers propose co-printing with bioactive molecules, for instance, growth factors, as an effective compensatory strategy for addressing the inherent lack of bioactivity in synthetic materials (Longoni et al., 2021).

While synthetic polymers offer tunable mechanical properties crucial for initial load bearing, a key challenge and novel perspective for “in motion” repair lies in designing these bioinks to not only match the dynamic mechanical properties of native T/L tissue but also to adapt over time (Xie et al., 2023). This includes developing synthetic bioinks with programmed viscoelasticity and fatigue resistance that can withstand continuous, complex loading cycles, crucial for robust functional restoration in active scenarios (Van Belleghem et al., 2020). Furthermore, strategies to mitigate their inherent bio-inertness and inflammatory potential under dynamic physiological conditions, such as incorporating mechano-responsive elements or anti-inflammatory agents within their structure, are essential for long-term integration and performance in motion (Shou et al., 2023).

#### 2.1.3 Cell sources and optimization

Tendon stem/progenitor cells (TSPCs), mesenchymal stem cells (MSCs), and induced pluripotent stem cells (iPSCs) represent primary cell sources for constructing T/L grafts, each with distinct advantages and limitations. TSPCs have ideal differentiation potential (Bi et al., 2007); MSCs offer ease of acquisition and immunomodulation but face potential immune rejection (Lohan et al., 2017; Huang et al., 2022); iPSCs provide large-scale autologous cell production but require optimized differentiation protocols (Farkhondeh et al., 2019; Haller et al., 2019). Current research emphasizes enhancing tenogenic differentiation via growth factors, mechanical stimuli, or gene editing (CRISPR/Cas9) (Hsu et al., 2019; Razavi et al., 2024), alongside establishing standardized allogeneic cell banks to manage variability and immunogenic concerns (Hazrati et al., 2022).

Variations in tenogenic differentiation efficiency, immunogenicity (particularly with allogeneic sources), ease of access, and ethical limitations are observed among different cell sources (Subramanian et al., 2017; Oh et al., 2019). Autologous cells, despite being free from immune issues, present limitations in availability, and their quality can be affected by the patient’s age or pathological state. Challenges associated with allogeneic cells, especially MSCs, include potential immune rejection and functional heterogeneity (Jiang and Xu, 2019); consequently, establishing standardized cell banks with good tenogenic potential is recognized as an important developmental direction (Liu et al., 2016). Differing views also exist regarding whether cells should be pre-differentiated towards the T/L lineage before printing, or if an inductive microenvironment should be provided within the printed construct to promote *in situ* differentiation (Xu et al., 2019; Tu et al., 2023). While optimizing tenogenic differentiation is a foundational goal, a novel and critical perspective for “in motion” T/L repair centers on selecting and engineering cell sources that are intrinsically robust and responsive to the dynamic mechanical cues present during movement (Shojaee et al., 2022). This involves not just achieving basic differentiation but also imbuing cells with enhanced mechanosensing and mechanotransduction capabilities, allowing them to actively participate in the adaptive remodeling of the graft under continuous, variable loads. Future efforts might explore specific genetic or epigenetic modifications, or novel pre-conditioning strategies, to prime cells for optimal survival, integration, and extracellular matrix production that specifically contributes to the dynamic resilience and long-term functional stability required in motion (Lai et al., 2023).

#### 2.1.4 Composite/functionalized bioinks

The development of multi-component hydrogel bioink systems has opened up new possibilities for enhancing printing accuracy, shape fidelity, and biological functionality (Mouser et al., 2020). In the composition of bioinks, the combination of natural and synthetic materials has been shown to enhance the stability and biocompatibility of bioinks. For example, a composite bioink made from methyl methacrylate-modified xanthan gum and gelatin exhibits excellent shear thinning (a property where viscosity decreases under applied shear stress, facilitating smooth extrusion) properties and biocompatibility, which makes it highly suitable for 3D bioprinting with superior printing performance and cell viability (Kozlowska et al., 2020). Moreover, the introduction of nanocomposites offers a new approach to enhancing bioinks by improving their viscosity, printability, and biocompatibility, thus showing significant potential in bone and cartilage tissue engineering (Rodríguez-Padrón et al., 2017). In practical applications, the optimization of bioinks involves not only material selection but also adjustments to printing parameters. For instance, studies have shown that by modifying the composition of bioinks and adjusting printing parameters, the shape fidelity and cell viability of printed embryonic stem cells can be significantly enhanced (Chen et al., 2010). Furthermore, the microscopic heterogeneity of bioinks has been shown to improve the quality of printed complex structures and cell viability, offering new strategies for high-resolution 3D printing (Park et al., 2022). Additionally, using enzyme-induced dynamic degradation methods can gradually release space without affecting cell activity, thereby promoting cell proliferation and the establishment of tissue function (Cianfanelli et al., 2015). The core objective of bioink design is to create a favorable microenvironment for cells. By optimizing the composition and structure of bioinks, it can provide the necessary biochemical and mechanical signals for cell proliferation and differentiation. For instance, using protein-rich bioinks can significantly enhance cell proliferation and responsiveness (Clarke et al., 2017). These methods ensure that the shape of the bioink is maintained and the printing accuracy is maintained during the printing process.

### 2.2 Structural biomimetic design and post-printing maturation strategies

Native T/L tissue possesses a complex multi-scale, hierarchical, and anisotropic (meaning its properties, like strength or elasticity, vary depending on the direction of measurement) structure, which is fundamental to its superior mechanical properties and physiological functions. 3D bioprinting offers the potential to accurately replicate this complex architecture (Ma et al., 2022; Altunbek et al., 2023).

#### 2.2.1 Multi-scale structural biomimicry

Mimicking the structural features of native tissue at molecular, nano, micro, and macro scales is a crucial goal for ideal T/L grafts (Tachibana et al., 2022). These features encompass the parallel alignment and crimped morphology of collagen fibers, the spindle-shaped and oriented distribution of cells, and the hierarchical assembly from fascicles to tendon bundles to the entire tendon (Laranjeira et al., 2017). Specifically, the tendon-to-bone enthesis displays a pore size gradient from 50 µm in the collagen-rich tendon region to approximately 300 µm in the mineralized fibrocartilage, with the elastic modulus increasing steeply from 200 MPa in tendon to 20 GPa in the bone region, and the osteochondral interface shows a mechanical stiffness increasing from approximately 0.02 MPa (superficial) to 6 MPa (calcified) and up to 15–20 GPa in bone (Khalak et al., 2025). Advanced 3D bioprinting techniques, including multi-nozzle/multi-material printing, microfluidic-assisted bioprinting, near-field direct writing, and hybrid melt-electrowriting, offer powerful tools for constructing T/L scaffolds that possess graded compositions, layered structures, and anisotropic mechanical properties (Altunbek et al., 2023). Computer-aided design (CAD), by integrating patient-specific MRI or CT imaging data, can facilitate patient-specific macroscopic anatomical reconstruction and guide printing paths to control the orientation of internal microstructures, a critical aspect for restoring mechanical transduction “in motion.”

While progress has been achieved in biomimicry at macro and some micro scales, a significant research gap and challenge remain in precisely controlling the orientation of individual cells, alongside the self-assembly and micro-alignment of extracellular matrix (especially collagen fibers) during the printing process (Wang et al., 2021). Such control is essential to achieve functional cell-matrix interactions and effective mechanical transduction, particularly within complex interface regions like the tendon/ligament-bone junction (enthesis).

While significant progress has been achieved in replicating the static multi-scale architecture, a novel perspective for in motion repair focuses on biomimetic designs that ensure structural integrity and dynamic functionality under repetitive and complex physiological loads (Chansoria et al., 2022). This goes beyond mere replication, aiming for architectures that exhibit programmed viscoelasticity, fatigue resistance, and adaptive remodeling capabilities, directly reflecting the demands of natural T/L tissue during continuous movement. Furthermore, the challenge of precisely controlling cell orientation and the self-assembly of extracellular matrix (especially collagen fibers) during printing gains a new dimension when considering the necessity for optimized mechanical transduction and load distribution at tissue interfaces (like the enthesis) during dynamic motion (Pan et al., 2008). These advanced biomimetic designs are paramount for recreating the complex interplay between structure and function essential for robust T/L repair in motion.

#### 2.2.2 Post-printing maturation culture and functionalization

Freshly printed bioconstructs frequently lack the sufficient mechanical strength and biological maturity necessary for direct implantation and withstanding physiological loads, making the post-printing *in vitro* maturation phase crucial. Bioreactors, through mimicking the *in vivo* physiological environment and particularly the mechanical stimulus environment (utilizing, for example, uniaxial/multiaxial tensile stretch, perfusion, torsion, or combined mechanical stimuli), can significantly promote cell proliferation, oriented differentiation, and the deposition and remodeling of extracellular matrix, especially Type I collagen (Gensler et al., 2024; Thangadurai et al., 2024). Furthermore, it has been highlighted that without functional vascular networks, the thickness of 3D bioprinted tissue constructs is limited to approximately 1 mm due to diffusional constraints, emphasizing the critical role of promoting angiogenesis, for instance, by encapsulating vascular endothelial growth factor (VEGF) in gelatin micro-particles to achieve controlled delivery for up to 3 weeks and enhance vascularization (Potyondy et al., 2021). This process, in turn, enhances the graft’s mechanical properties, such as elastic modulus, ultimate tensile strength, and toughness, as well as its biological function (Machour et al., 2022). Sequential or targeted addition of growth factors (including TGF-β1/β3, GDF-5/6/7, CTGF, and FGF) to the culture medium has also been demonstrated as an effective method to promote T/L tissue maturation (Zhang et al., 2021).

Determining the optimal parameter combination for mechanical stimulation—encompassing type, frequency, magnitude, duration, loading pattern, and timing of initiation—presents an ongoing challenge, as these parameters are not currently standardized and may vary depending on the cell type used, bioink material, and graft design, thus requiring extensive experimental exploration (Pardo et al., 2022). A further debatable issue is the extent to which *in vitro* maturation should proceed before implantation. Excessive *in vitro* maturation carries the risks of increased culture time, cost, and potential contamination, and may also render the graft too “rigid,” thereby limiting its ability to undergo adaptive remodeling within the complex *in vivo* microenvironment post-implantation (Ng et al., 2020).

While bioreactors significantly promote initial maturation, a novel perspective for in motion repair emphasizes the development of tailored and patient-specific maturation protocols that closely mimic the complex, dynamic, and multi-directional loading conditions encountered during actual movement (Grillner et al., 2020). This pre-conditioning aims to prime the graft for immediate functionality under dynamic physiological loads, not just static strength. Furthermore, the ongoing debate regarding the optimal extent of *in vitro* maturation before implantation gains new relevance here: the challenge lies in striking a balance that ensures sufficient mechanical competence for early loading (Ménézo et al., 2013), while preserving the graft’s inherent capacity for adaptive remodeling and integration within the dynamic in motion microenvironment post-implantation, ultimately aiming for long-term functional congruence with native tissues during activity.

#### 2.2.3 Emerging and convergent strategies

Beyond the direct biomimicry of structural features and post-printing maturation, the future of T/L tissue engineering may lie in convergent strategies inspired by broader advances in biofabrication. These approaches could address persistent challenges in achieving full biological functionality.

One promising direction is the adoption of hybrid or “bottom-up” assembly strategies. This involves combining 3D printing with self-organizing biological units. For instance, precisely arranged micro-cavities can be printed to guide the self-assembly of cell spheroids into structured tissues, offering enhanced control over local cell density and micro-architecture (Daly and Kelly, 2019). Similarly, integrating organoid technology with bioprinting allows for the creation of more physiologically relevant microenvironments that promote complex cellular interactions and functions (Hu et al., 2025).

Another significant hurdle for engineering large-scale grafts is vascularization. An emerging strategy tackles this by first assembling hundreds of thousands of high-density “organ building blocks” to form a living matrix, followed by printing a perfusable vascular network directly within this construct (Zhang et al., 2020). This method shows potential for rapidly creating patient-specific, vascularized tissues (Ma et al., 2019). Furthermore, novel printing modalities, such as pre-set extrusion bioprinting, are being developed to better control the deposition of multiple materials and cell types simultaneously, which is critical for fabricating heterogeneous structures like the complex tendon-bone junction (Kang et al., 2018). These convergent strategies represent exciting future avenues for creating the next-generation of truly functional T/L grafts.

From an in motion perspective, these approaches offer a novel paradigm for engineering grafts that are not merely structurally sound, but are also inherently designed for dynamic biomechanical integration and adaptive performance within the complex, variable loading environment of active movement (Feller et al., 2017). For instance, hybrid assembly and organoid technologies enable the creation of microenvironments that promote more physiologically relevant cellular interactions and matrix remodeling under dynamic stimuli (Kim and Kim, 2020). Furthermore, advanced vascularization strategies are crucial for sustaining long-term cellular viability and metabolic function under the heightened demands of motion, while novel multi-material printing modalities are key to fabricating interfaces that can efficiently and robustly transmit complex loads encountered in motion (Krishnan et al., 2014). These collective efforts aim to ensure the graft’s resilience, adaptive capacity, and seamless functional restoration throughout a patient’s dynamic life.

## 3 Discussion

Significant advancements in 3D bioprinting—encompassing bioink development, cell application strategies, structural biomimetic design, and post-printing maturation culture—have created unprecedented opportunities for constructing functional, patient-specific tendon/ligament grafts. This technology holds particular promise for addressing the shortcomings of traditional treatments in restoring complex functions “in motion,” offering new hope for T/L injury repair. However, successfully translating this technology from bench to bedside still faces numerous challenges and unresolved scientific questions.

### 3.1 Summary of major progress

3D bioprinting allows researchers to control the macro-morphology and micro-architecture of T/L grafts with unprecedented precision, and to integrate cells and bioactive factors, thereby constructing substitutes that more closely resemble native tissues. Bioinks are evolving from single-component to composite and functionalized formulations (Amaral et al., 2021); cell sources are expanding from traditional adult cells to stem cells and genetically engineered cells (Chae et al., 2022); printing strategies are progressing from simple structures to complex biomimetic designs (Loukelis et al., 2024); and *in vitro* maturation methods are shifting from static culture to functionalization culture under dynamic mechanical stimulation (Hull et al., 2021). These collective advancements are driving 3D bioprinted T/L grafts towards functionalization and personalization (Duan, 2016).

Furthermore, The development of smart bioinks has been particularly rapid, with some studies utilizing them to achieve responsive drug release cued by the *in vivo* microenvironment (Maan et al., 2022), while others have achieved self-adapting material properties through dynamic crosslinking regulation. Further advancements include the use of smart designs for hybrid bioprinting of scalable and viable tissue constructs and the fabrication of complex tissue scaffolds with *in situ* homogeneously mixed bioinks using advanced portable biopen technology (Schwab et al., 2020). Alongside smart bioinks, 4D bioprinting is emerging as a transformative strategy, focusing on post-print dynamic transformation, mechanical adaptability (Gao et al., 2016), and programmable responses, including biocompatible composite hydrogels with on-demand swelling-shrinking properties and multimaterial 3D and 4D bioprinting for heterogeneous constructs. Additionally, artificial intelligence (AI) is playing an increasingly pivotal role in AI-assisted design (J et al., 2022), where machine learning optimizes printing parameters, AI assists in modeling, and print paths are optimized, leading to more efficient and precise personalized treatment regimens. For a more comprehensive and extensive list of relevant studies applications, please refer to Table 1.

**TABLE 1:** Emerging research directions and innovative strategies in 3D bioprinting.

Research direction	Representative studies	Core Content/Highlights
Smart Bioinks	Kim and Cho (2024), Cong and Zhang (2025), Schwab et al. (2020), Zhu et al. (2024), Nandakumar et al. (2024), Bhattacharyya et al. (2023), Gao et al. (2023), Maan et al. (2022)	Responsive drug release, bio-adaptive materials, dynamic cross-linking regulation
4D Bioprinting	Lai et al. (2024), Wang et al. (2023a), Amukarimi et al. (2022), Jensen et al. (2025), Zhang et al. (2025a), Gao et al. (2016), Agarwal et al. (2024), Shi et al. (2024), Chen et al. (2024a)	Post-print dynamic transformation, mechanical adaptability, programmable response
AI-assisted Design	Zhang et al. (2025b), Jean et al. (2022), Chen et al. (2024b), Bhardwaj et al. (2024), Jo et al. (2023)	Machine learning optimizes printing parameters, AI assists in modeling, and print paths are optimized

### 3.2 Current research gaps

#### 3.2.1 Perfect match and dynamic response of mechanical properties

Achieving an initial mechanical strength, anisotropy, viscoelasticity, and long-term dynamic mechanical behavior (e.g., fatigue resistance, creep, stress relaxation) in printed grafts that closely match native T/L tissue—to withstand complex, sustained, and variable mechanical loads “in motion”—remains a core challenge. This requires breakthroughs not only in materials science but also a deeper understanding of T/L biomechanics and injury repair mechanisms (Freeman and Kelly, 2017; Li et al., 2022).

#### 3.2.2 Vascularization and innervation

For larger-sized 3D bioprinted T/L grafts, the long-term survival and function of internal cells heavily depend on rapid and effective vascularization. Although some strategies have been attempted (e.g., co-culturing endothelial cells, pre-fabricating vascular networks, embedding pro-angiogenic factors), achieving a functional, stable capillary network that eventually anastomoses with the host vascular system remains a formidable challenge (Cui et al., 2016a; Gold et al., 2021). For example, in pre-fabricating vascular networks, approaches include using sacrificial bioinks to print perfusable channels within the main construct, which are then removed after printing is complete to form a vascular network (J et al., 2022), as demonstrated in strategies aiming to create vascularized tissue models. Furthermore, embedding pro-angiogenic factors can involve incorporating biologically inspired smart release systems within 3D bioprinted scaffolds to promote vascularized tissue regeneration (Cui et al., 2016b). Innervation, crucial for proprioception, coordinated movement, and tissue homeostasis, is currently a very nascent area of research in this context (Mörö et al., 2022). Despite the limited current research, potential strategies for achieving functional innervation could include borrowing from nerve tissue engineering to explore co-culturing with neural stem cells, integrating neurotrophic factors into the bioink, or utilizing conductive biomaterials to guide neurite outgrowth and establish functional connections. These promising avenues represent crucial future research hotbeds, as successful innervation would profoundly enhance graft functionality, enabling more natural proprioception, coordinated movement, and overall tissue long-term homeostasis within dynamic “in motion” scenarios.

#### 3.2.3 Interface tissue engineering

The tendon/ligament-bone interface (enthesis) is a transitional zone with a complex graded structure (from tendon/ligament to uncalcified fibrocartilage, calcified fibrocartilage, and then bone) and unique mechanical properties. Its functional regeneration is critical for stable graft anchorage and effective mechanical load transmission. Reconstructing this multi-tissue, multi-phase interface using 3D bioprinting remains extremely challenging (Altunbek et al., 2023; Du et al., 2023).

#### 3.2.4 Immunomodulation and inflammatory response

Even when using autologous cells, the biomaterials themselves, the printing process, and surgical trauma can trigger host immune and inflammatory responses. Modulating the immune microenvironment at the implantation site to suppress destructive inflammation and promote constructive tissue remodeling rather than fibrosis or heterotopic ossification is key to ensuring long-term graft success (Du et al., 2024).

### 3.3 Different Schools of thought or controversies

#### 3.3.1 “Structure-first” vs “cell/bioactivity-first”

There are differing emphases regarding the core design philosophy for 3D bio-printed grafts. Some scholars emphasize perfectly replicating the multi-scale complex structure of native T/L tissue through precise printing techniques, believing structure is the foundation of function (Altunbek et al., 2023). Others argue that providing the appropriate cell types and a bioactive microenvironment capable of inducing cell differentiation and matrix production is more critical, trusting that cells, under proper guidance, will actively remodel and form a functional structure (Yang et al., 2022b). An ideal strategy likely involves an organic combination of both.

#### 3.3.2 *In Vitro* maturation vs in vivo remodeling

There are varied opinions on the extent to which 3D bioprinted T/L grafts should be matured *in vitro* before implantation. Some advocate that grafts should achieve mechanical properties and biological maturity comparable to native tissue through *in vitro* mechanical stimulation before implantation, ensuring they can withstand early physiological loads (Sun et al., 2020; Yang et al., 2022a). However, excessive or prolonged *in vitro* maturation not only increases culture costs, time, and potential contamination risks but might also render the graft too “static,” limiting its capacity for adaptive remodeling within the complex *in vivo* microenvironment post-implantation (Chen et al., 2020). Finding the optimal balance between *in vitro* pre-maturation and *in vivo* dynamic remodeling is an important future research direction.

### 3.4 Challenges faced

#### 3.4.1 Scale-up and standardized production

Most current 3D bioprinting T/L research is still at the laboratory stage. To achieve clinical application, reproducible, cost-effective, and scalable production compliant with Good Manufacturing Practice (GMP) standards must be addressed, including standardized bioink preparation, precise control of the printing process, and quality monitoring (Stanco et al., 2020).

#### 3.4.2 Regulatory approval pathways

The regulatory approval pathway for 3D bioprinted T/L grafts, as novel medical products combining cells, biomaterials, and engineered structures (potentially classified as Advanced Therapy Medicinal Products (ATMPs) or Tissue Engineered Medical Products (TEMPs)), is not yet fully clear or harmonized globally. This requires collaborative efforts from regulatory agencies, researchers, and industry to establish clear evaluation criteria and approval processes (Ying et al., 2018).

#### 3.4.3 Lack of long-term in vivo studies

The vast majority of studies are still limited to *in vitro* experiments or small animal models, which cannot fully replicate the complex pathophysiology of human T/L injuries or the mechanical loads experienced in motion. There is an urgent need for long-term *in vivo* functional evaluations in large animal models (such as canines, ovines, porcines, or non-human primates) that more closely mimic human physiological conditions, to validate the safety and efficacy of 3D bioprinted grafts under realistic mechanical loading (Gold et al., 2021).

### 3.5 Ethical considerations

#### 3.5.1 Ethics of cell sourcing

The rapid advancement of 3D bioprinting technology, particularly for patient-specific grafts, necessitates a robust consideration of various ethical dimensions that extend beyond purely translational challenges. Firstly, the ethics of cell sourcing present significant considerations. While autologous cells offer immune compatibility, their limited availability and variability based on patient age or pathological state are notable (Bell et al., 2017). The increasing reliance on induced pluripotent stem cells (iPSCs) provides a scalable autologous source, yet raises questions concerning the ethical implications of their derivation and manipulation (Carelli et al., 2022), echoing some debates associated with embryonic stem cells. Similarly, the use of allogeneic cell banks, particularly mesenchymal stem cells, while addressing scalability, introduces challenges related to potential immune rejection and the ethical complexities of donor consent, anonymity, and commercialization of biological materials (Parekkadan and Milwid, 2010).

#### 3.5.2 Data privacy and informed consent in personalized therapy

Ensuring data privacy and patient informed consent is paramount in the realm of personalized therapy. The design and fabrication of patient-specific grafts heavily rely on sensitive medical imaging data (e.g., MRI, CT) and potentially genetic information (Chen et al., 2022). Robust frameworks are required to protect this highly personal data from breaches and misuse. Furthermore, given the innovative and experimental nature of 3D bioprinting in clinical applications, comprehensive informed consent processes must clearly articulate the potential risks, benefits, and uncertainties to patients (Hsu and Jiang, 2019), ensuring their understanding and voluntary participation in such advanced therapeutic regimens.

#### 3.5.3 Cost, accessibility, and healthcare equity

The potential cost and accessibility issues associated with such a highly advanced technology pose significant ethical dilemmas regarding healthcare inequalities (Lakdawala et al., 2013). The development and standardized production of 3D bioprinted T/L grafts are likely to be expensive, raising concerns about equitable access for all patients who could benefit. Addressing how to mitigate these cost barriers and ensure that these life-changing therapies do not exacerbate existing disparities in healthcare access is a critical ethical challenge that requires proactive policy development and collaborative efforts among researchers, industry, and healthcare systems (Arasaradnam, 2001).

#### 3.5.4 Regulatory and safety considerations for gene-edited cells

The growing incorporation of gene-edited cells, such as iPSCs modified via CRISPR/Cas9, into 3D bioprinted grafts introduces specific clinical risks that demand robust regulatory oversight (Huo et al., 2022). A primary concern is the potential for off-target effects, where unintended genomic changes could lead to unforeseen consequences, altered cell behavior, immunogenicity by presenting novel antigens, or even tumorigenicity through disruption of regulatory pathways (Tang et al., 2019). Addressing these intricate safety profiles necessitates a stringent regulatory framework. Bodies like the European Medicines Agency and its Committee for Advanced Therapies establish rigorous guidelines for Advanced Therapy Medicinal Products, requiring extensive preclinical safety evaluations (including genome integrity, immunogenicity, and tumorigenic potential), strict manufacturing quality control, and meticulously designed clinical trials with long-term follow-up to ensure safety and efficacy before clinical translation (Vanessa et al., 2021).

## 4 Potential future developments in the field

**FIGURE 2:**
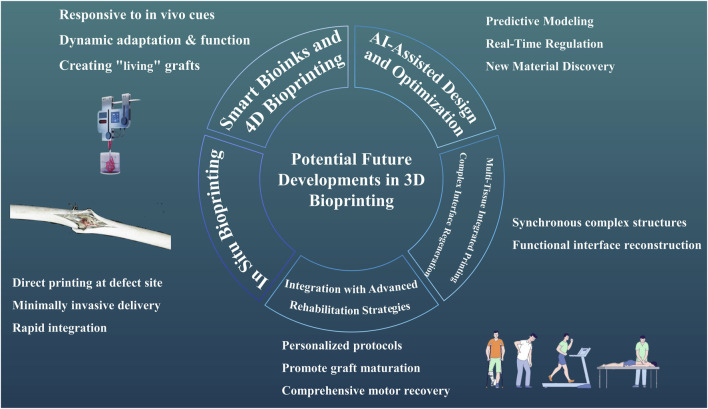
Potential Future Developments in 3D Bioprinting A picture illustrates potential future developments in 3D bioprinting for tendon/ligament (T/L) repair, encompassing smart bioinks and 4D bioprinting, *in situ* bioprinting, AI-assisted design and optimization, multi-tissue integrated printing, and integration with advanced rehabilitation strategies.

Looking ahead, several transformative areas are poised to shape the future of 3D bioprinting for T/L repair, encompassing innovations from smart materials to advanced integration strategies (Figure 2).

### 4.1 Smart bioinks and 4D bioprinting

Developing smart bioinks capable of responding to *in vivo* microenvironmental cues (e.g., pH, temperature, specific enzymes, mechanical signals) by undergoing programmed deformation, releasing bioactive substances, or altering their mechanical properties, combined with 4D bioprinting strategies (i.e., 3D printed structures that change shape or function over time in a pre-programmed manner), promises to create “living” grafts that can better adapt to and participate in the dynamic repair process “in motion” (Maan et al., 2022; Wang et al., 2023a; Abolhassani et al., 2025).

### 4.2 *In Situ* bioprinting

Exploring techniques for direct 3D bioprinting at the site of T/L defect in a patient, delivering printing devices minimally invasively to the injury site, and potentially using the patient’s own cells and tissue fluids as part of the printing material This could minimize surgical trauma and promote rapid integration of the graft with host tissues (Macadam et al., 2022; Wang et al., 2023b).

### 4.3 AI-assisted design and optimization

Utilizing artificial intelligence (AI) and machine learning algorithms, based on extensive experimental data and clinical imaging information, to assist in optimizing bioink formulations, printing parameters, 3D graft structural design, and even predicting graft performance and remodeling processes *in vivo*, thereby enabling more efficient and precise personalized treatment regimens (Ning et al., 2023; Chen et al., 2024b). Specifically, AI/machine learning can be used not only to optimize printing pathways and parameters but also for: Predictive Modeling: Predicting the mechanical properties and biological functions of the final graft based on bioink composition, cell type, and culture conditions; Real-Time Regulation: Integrating sensors within bioreactors to analyze tissue maturation status in real-time and dynamically adjust mechanical or chemical stimulation protocols; New Material Discovery: Assisting in the design and screening of novel bioink formulations with ideal properties by analyzing vast amounts of literature and experimental data.

### 4.4 Multi-tissue integrated printing and complex interface regeneration

Developing technologies capable of synchronously printing complex multi-tissue structures containing various cell types (e.g., fibroblasts, chondrocytes, osteoblasts) and different matrix components, to achieve functional reconstruction of complex interfaces like the tendon/ligament-bone junction in a single printing process (Murali and Parameswaran, 2024).

### 4.5 Integration with advanced rehabilitation strategies

Future research should increasingly focus on combining post-implantation of 3D bio-printed grafts with personalized, biomechanics-informed advanced rehabilitation protocols. By precisely controlling the post-operative mechanical environment, this synergy can promote functional maturation of the graft and comprehensive recovery of the patient’s motor abilities (Daikuara et al., 2021; Rahimnejad et al., 2021).

## 5 Conclusion

3D bioprinting is poised to revolutionize tendon and ligament (T/L) repair, offering a powerful alternative to traditional grafts by fabricating patient-specific, bioactive, and biomimetic constructs essential for functional recovery “in motion.” Despite significant advances in bioinks, cell integration, and structural design, the field still faces critical challenges, including the need to perfectly match dynamic mechanical properties, ensure long-term viability through vascularization and innervation, and overcome hurdles in scalable manufacturing and clinical translation. Looking forward, emerging technologies like smart materials, 4D bioprinting, and AI-assisted design will be key to surmounting these obstacles. Ultimately, the goal of 3D bioprinting is to shift the treatment paradigm from simple repair to true biological and functional regeneration, enabling patients to fully return to their dynamic lives.
